# Prostate-specific membrane antigen targeted organic semiconducting polymer nanoparticles for enhanced photothermal therapy of prostate cancer

**DOI:** 10.3389/fimmu.2025.1688048

**Published:** 2026-01-09

**Authors:** Zhongji Jiang, Xun Zhang, Gaohaer Kadeerhan, Jin Zhang, Jiali Jin, Weijing Hu, Wenmin Guo, Hong Guo, Dongwen Wang

**Affiliations:** 1 School of Medicine, Southern University of Science and Technology, Shenzhen, Guangdong, China; 2 Department of Urology, National Cancer Center/National Clinical Research Center for Cancer/Cancer Hospital & Shenzhen Hospital, Chinese Academy of Medical Sciences and Peking Union Medical College, Shenzhen, Guangdong, China; 3 Department of Materials Science and Engineering, Southern University of Science and Technology, Shenzhen, Guangdong, China; 4 Central Laboratory & Shenzhen Key Laboratory of Epigenetics and Precision Medicine for Cancers, National Cancer Center/National Clinical Research Center for Cancer/Cancer Hospital and Shenzhen Hospital, Chinese Academy of Medical Sciences and Peking Union Medical College, Shenzhen, Guangdong, China; 5 First College of Clinical Medicine, Shanxi Medical University, Taiyuan, Shanxi, China; 6 Department of Urology, First Hospital of Shanxi Medical University, Taiyuan, Shanxi, China; 7 Southern University of Science and Technology, Shenzhen, Guangdong, China

**Keywords:** NIR-II, organic semiconducting polymer, photothermal therapy (PTT), prostate cancer, prostate-specific membrane antigen (PSMA), tumor microenvironment (TME)

## Abstract

Photothermal therapy (PTT) in the second near-infrared window (NIR-II, 1000–1700 nm) enables deep-tissue penetration and reduced off-target damage, offering a promising approach for localized cancer ablation. A major challenge, however, lies in achieving efficient and tumor-specific accumulation of photothermal agents. In this study, we developed a prostate-specific membrane antigen (PSMA)-targeted NIR-II photothermal nanoplatform based on an organic semiconducting polymer (OSP_12_). The OSP_12_ core was encapsulated with DSPE-PEG-Mal and covalently conjugated with ACUPA, a high-affinity PSMA ligand, to generate PSMA-OSP_12_ nanoparticles (NPs). These nanoparticles exhibited strong NIR-II fluorescence emission and high photothermal conversion efficiency under 808 nm excitation; notably, at 1.0 W/cm^2^ for 5 min the maximum solution temperature reached 77.3°C, and the particles showed excellent photothermal stability, retaining >90.0% of their peak heating performance after five on/off irradiation cycles. Owing to their enhanced targeting capability and robust photothermal stability, PSMA-OSP_12_ NPs enabled effective photothermal ablation of PSMA-positive prostate tumors with minimal systemic toxicity *in vivo*. Collectively, our findings demonstrate that PSMA-OSP_12_ NPs constitute a potent and precise NIR-II photothermal nanoplatform for prostate cancer treatment.

## 1 Introduction

Prostate cancer (PCa) is one of the most commonly diagnosed malignancies among men and remains a leading cause of cancer-related deaths worldwide (1–3). The deep anatomical location of the prostate, surrounded by critical structures such as neurovascular bundles, the bladder, and rectum, presents considerable challenges for complete tumor resection (4–6). Although radical prostatectomy is a standard treatment for localized PCa, the complexity of pelvic anatomy makes achieving negative surgical margins without damaging adjacent tissues technically demanding, even for experienced surgeons (7). The advent of robotic-assisted surgical systems, such as the da Vinci^®^ platform, has improved surgical precision; however, intraoperative tumor localization largely relies on visual and tactile feedback, which are often inadequate (8, 9). In addition, despite advances in treatment modalities, including surgery, radiotherapy, and hormone therapy, recurrence and metastasis remain major clinical challenges, especially in advanced stages (4, 10). These limitations underscore the need for real-time, fluorescence-guided imaging strategies to enhance intraoperative tumor delineation, minimize residual disease, and improve patient outcomes.

Photothermal therapy (PTT) has emerged as a promising minimally invasive modality for cancer treatment (11, 12). PTT utilizes photothermal agents (PTAs) to convert absorbed light energy into heat, inducing localized hyperthermia that can ablate tumor tissues (11, 13, 14). Compared to conventional therapies, PTT offers several advantages, including high spatial-temporal precision, minimal invasiveness, and the potential to stimulate anti-tumor immune responses (11, 14). However, the clinical translation of PTT has been hindered by the limited tissue penetration depth of near-infrared-I (NIR-I, 650–900 nm) light, which restricts its efficacy in treating deep-seated tumors like prostate cancer.

To overcome this limitation, the second near-infrared window (NIR-II, 1000–1700 nm) has garnered significant attention for biomedical applications (15–17). NIR-II light offers deeper tissue penetration, reduced photon scattering, and higher maximum permissible exposure (MPE) compared to NIR-I light, making it more suitable for treating deep tumors (18–22). Notably, the classification of NIR-II probes is defined by their emission wavelength, not the excitation source. As first demonstrated by Dai’s group in 2009, semiconducting single-walled carbon nanotubes exhibited NIR-II emission (1100–1700 nm) under 808 nm excitation, achieving the first *in vivo* NIR-II imaging in mice (23). This pioneering work established that efficient NIR-II emission can be realized with NIR-I excitation, a principle widely adopted in subsequent studies employing 808 nm excitation for >1000 nm emission (24–26). Consequently, the development of NIR-II-absorbing PTAs with high photothermal conversion efficiency (PCE), excellent biocompatibility, and tumor-targeting capabilities is critical for advancing PTT in clinical settings. High-temperature PTT (>50°C) can effectively ablate tumor cells but carries the risk of damaging surrounding normal tissues, whereas mild-temperature PTT (42–45°C) is relatively safer but may induce heat shock protein (HSP) upregulation, leading to thermotolerance and reduced efficacy. Therefore, there is an urgent need to achieve precise targeting in photothermal therapy to ensure both safety and therapeutic effectiveness.

Organic semiconducting polymers (OSPs) have emerged as attractive candidates for constructing NIR-II PTAs due to their tunable optical properties, good biocompatibility, and structural versatility (27–29). Recent studies have demonstrated that incorporating weak electron donors into the polymer backbone can suppress vibrational relaxation, thereby enhancing NIR-II absorption and photothermal performance (29–31). For instance, OSP_12_ nanoparticles (NPs), a newly developed semiconducting polymer, exhibits remarkable NIR-II absorption and a broad tail extending to 1200 nm. Additionally, it achieves a high PCE of 45.25%, attributed to its optimized molecular design that minimizes non-radiative energy loss. These properties render OSP_12_ NPs a promising core material for constructing efficient NIR-II PTAs (29).

Despite the favorable photophysical properties of OSP_12_ NPs, its lack of inherent tumor-targeting capability limits its therapeutic efficacy and may lead to off-target effects. To address this challenge, active targeting strategies have been employed to enhance the accumulation of PTAs in tumor tissues. Prostate-specific membrane antigen (PSMA) is a transmembrane glycoprotein that is highly overexpressed in prostate cancer cells, particularly in advanced and metastatic stages, while exhibiting limited expression in normal tissues (32, 33). This makes PSMA an ideal target for selective delivery of therapeutic agents to prostate tumors (34–37). Although PSMA-617 and PSMA-11 both share the glutamate-urea-lysine pharmacophore, their physicochemical properties limit further adaptation into nanoprobe platforms: PSMA-617 is highly hydrophobic and lacks suitable functional groups for conjugation, while PSMA-11 incorporates bulky chelating moieties that can hinder surface modification and compromise probe assembly. Therefore, we selected ACUPA, a small-molecule ligand that specifically binds to the extracellular domain of PSMA and enables efficient site-specific conjugation via thiol–maleimide chemistry, thereby facilitating the targeted delivery of conjugated nanoparticles (6, 38–41).

In our previous study, we established that PSMA-OSP_12_ NPs possess excellent tumor-targeting capability and high-performance NIR-II fluorescence emission, enabling accurate molecular imaging and diagnostic applications in prostate cancer (25). Building upon these findings, the present work extends the application of PSMA-OSP_12_ NPs from imaging to therapy, with a particular focus on their photothermal functionality. By systematically evaluating their physicochemical properties, photothermal conversion efficiency, targeting specificity, *in vitro* cytotoxicity, *in vivo* therapeutic efficacy, and biosafety, we demonstrate that PSMA-OSP_12_ NPs provide not only precise tumor ablation but also potential immunoregulatory benefits. This continuity highlights the translational value of PSMA-OSP_12_ NPs as an integrated nanoplatform for both diagnosis and treatment of prostate cancer.

## 2 Materials and methods

### 2.1 Synthesis of PSMA-targeted photothermal nanoparticles

The photosensitizer OSP_12_ was synthesized according to our previously reported method (29). To construct PSMA-targeted nanoparticles, OSP_12_ (5 mg) and DSPE-PEG-Mal (15 mg, MW 3400) were co-dissolved in tetrahydrofuran (THF, 2 mL), followed by slow injection into deionized water (10 mL) under vigorous stirring. The organic solvent was evaporated by rotary evaporation, and the resulting OSP_12_-loaded nanoparticles were collected by ultrafiltration (MWCO: 10 kDa). For PSMA targeting, the thiol-modified ligand ACUPA-SH was added (molar ratio: 1.5:1 to maleimide groups) and reacted overnight at 4°C to obtain PSMA-OSP_12_ NPs via thiol–maleimide click chemistry (25).

### 2.2 Characterization of nanoparticles

Photothermal performance was evaluated by irradiating nanoparticle dispersions (0–0.2 mg/mL) with an 808 nm NIR laser (MDL-808-5W, 1.0 W/cm^2^, 5 min) while recording real-time temperature changes using a FLIR thermal camera.

#### 2.2.1 Bioinformatic analysis and molecular docking

FOLH1 (PSMA) gene expression data in prostate cancer (PRAD) and normal tissues were extracted from The Cancer Genome Atlas (TCGA) and analyzed using GEPIA2. Protein expression and subcellular localization of PSMA were retrieved from The Human Protein Atlas. The protein structure of PSMA was downloaded from the RCSB PDB database (PDB ID: 5O5U), and the ligand ACUPA was constructed using Chem3D. Molecular docking was performed using AutoDock Vina 1.1.2. Grid parameters were set to cover the extracellular catalytic domain of PSMA. The docking pose with the lowest binding energy was selected for visualization and interaction analysis using PyMOL.

### 2.3 *In vitro* cytotoxicity and live/dead staining

LNCaP prostate cancer cells (PSMA-positive) were cultured in RPMI-1640 medium supplemented with 10% fetal bovine serum and 1% penicillin/streptomycin. Cells were seeded into 96-well plates (1 × 10^4^ cells/well) and treated with OSP_12_ NPs or PSMA-OSP_12_ NPs (0–0.2 mg/mL) for 24 h, followed by laser irradiation (808 nm, 1.0 W/cm^2^, 5 min) where applicable. Cell viability was assessed using a standard Cell Counting Kit-8 (CCK-8) assay. For live/dead staining, treated cells were incubated with Calcein-AM (2 μM) and propidium iodide (PI, 4 μM) for 20 min and imaged with a confocal fluorescence microscope (Zeiss LSM 880, Zeiss, Germany).

### 2.4 *In vivo* photothermal therapy in 22Rv1 tumor model

Male BALB/c nude mice (5–6 weeks old) were subcutaneously inoculated with 5 × 10^6^ 22Rv1 cells into the right flank. When tumors reached approximately 100 mm³, the mice were randomly assigned into six groups (n = 4): PBS, PBS + laser, OSP_12_ NPs, OSP_12_ NPs + laser, PSMA-OSP_12_ NPs, and PSMA-OSP_12_ NPs + laser. Nanoparticles were administered via tail-vein injection (100 μL, 2 mg/mL).

Before laser irradiation, mice were anesthetized using 2–3% isoflurane inhalation anesthesia delivered through a calibrated vaporizer. At 24 hours post-injection, tumors were exposed to an 808 nm laser (1.0 W/cm^2^, 5 min). Surface skin temperatures were recorded using an infrared thermal imaging camera.

Tumor volumes and body weights were monitored every other day for 14 days. Tumor volume was calculated using the formula: V = (length × width^2^)/2.

On Day 14, mice were euthanized under deep isoflurane anesthesia (3–4% inhalation) followed by cervical dislocation, in accordance with institutional humane endpoint guidelines. This method ensures rapid, painless, and ethically compliant euthanasia consistent with international standards for laboratory animal care.

Tumors were then collected for imaging and further analysis. All animal procedures were reviewed and approved by the Institutional Animal Care and Use Committee (IACUC) of TOPGM under approval number TOPGM-IACUC-2023-0111.

### 2.5 Histological and biosafety evaluation

To comprehensively assess the *in vivo* biosafety of PSMA-OSP_12_ NPs, both histopathological and hematological evaluations were performed. On Day 14 post-treatment, mice were sacrificed under anesthesia, and major organs, including the heart, liver, spleen, lung, and kidney, were harvested for histological examination. Tissues were fixed in 4% paraformaldehyde, dehydrated, embedded in paraffin, sectioned into 4 μm slices, and stained with hematoxylin and eosin (H&E) following standard protocols. The stained sections were examined under a light microscope to evaluate tissue morphology and to identify any pathological changes such as inflammatory infiltration, hemorrhage, necrosis, or cellular atypia.

For systemic toxicity assessment, blood samples were collected via retro-orbital bleeding prior to sacrifice. Routine hematology and serum biochemical analyses were performed using an automated hematology analyzer and a clinical chemistry analyzer (BC-2800Vet, Mindray, China), respectively.

### 2.6 Statistical analysis

All data are expressed as mean ± standard deviation (SD). Statistical analyses were performed using GraphPad Prism 9.0 (GraphPad Software, USA). One-way ANOVA was used for multi-group comparisons, and unpaired two-tailed Student’s t-test for two-group comparisons. Tumor growth and body weight changes were analyzed by two-way repeated measures ANOVA. P < 0.05 was considered statistically significant. Statistical significance is indicated as p < 0.05 (*), p < 0.01 (**), p < 0.001 (***) and p < 0.0001 (****).

## 3 Results and discussion

### 3.1 Design, and synthesis of PSMA-targeted NIR-II photothermal nanoparticles

**Figure 1:**
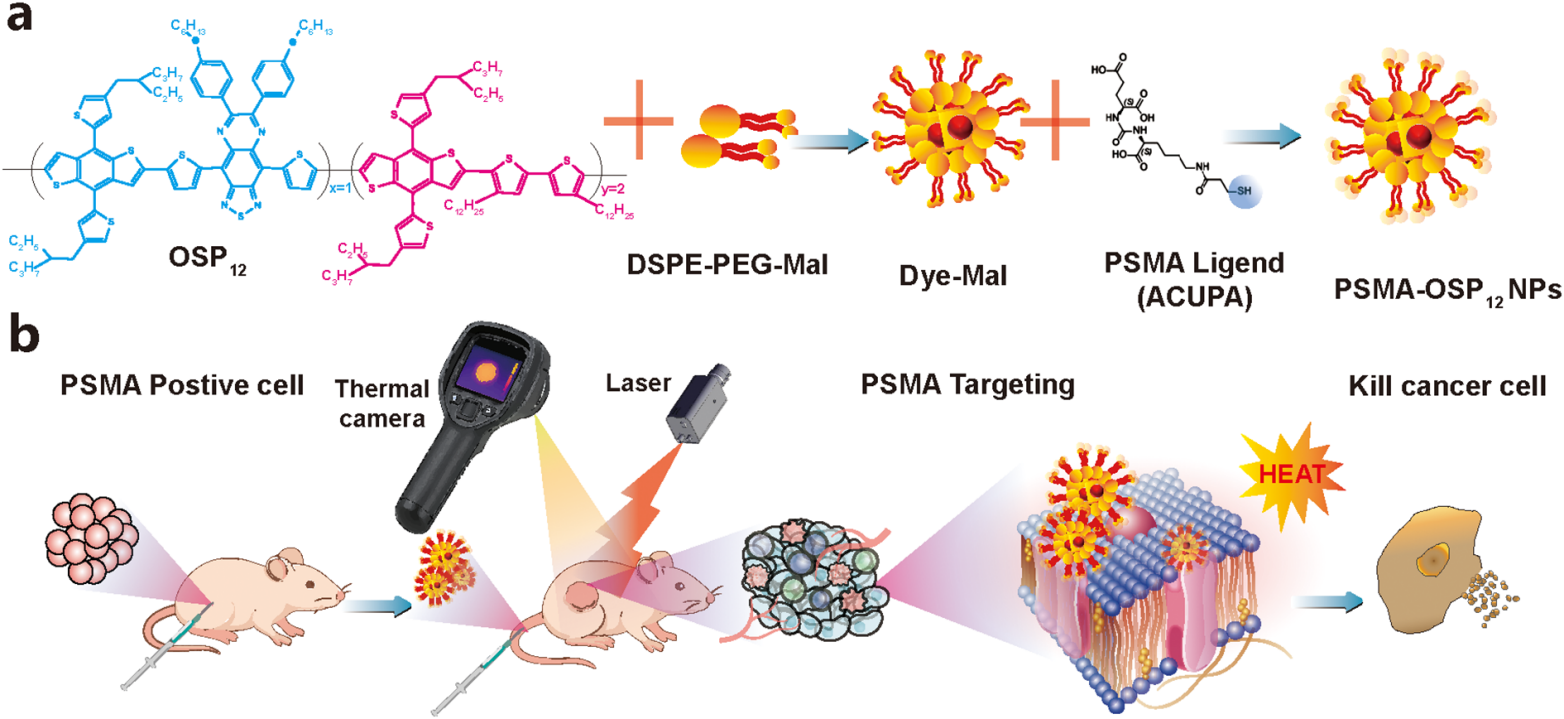
Schematic illustration of the design and therapeutic strategy of PSMA-targeted NIR-II photothermal agents. **(a)** PSMA-targeted photothermal agent (PSMA-OSP_12_ NPs) was synthesized by encapsulating NIR-II photosensitizer OSP_12_ with DSPE-PEG-Mal, followed by conjugation with a PSMA-targeting ligand. **(b)** 22Rv1 tumor-bearing mice were intravenously injected with PSMA-OSP_12_ NPs. Upon 808 nm laser irradiation (1.0 W/cm^2^, 5 min), significant tumor heating was observed via thermal imaging, enabling precise photothermal ablation of PSMA-positive tumors.

To enable efficient prostate cancer-specific PTT, we developed a PSMA-targeted NIR-II photothermal nanoplatform by integrating molecular design, targeted delivery, and photothermal functionality. The core photosensitizer, OSP_12_, a semiconducting polymer with suppressed vibrational relaxation and enhanced NIR-II absorption, was co-assembled with DSPE-PEG-Mal via nanoprecipitation to form stable polymeric nanoparticles. Subsequently, the PSMA-targeting ligand ACUPA-SH was covalently conjugated to the maleimide termini through thiol–maleimide Michael addition chemistry, yielding the final nanostructure denoted as PSMA-OSP_12_ NPs (**Figure 1a**).

The schematic illustration (**Figure 1b**) outlines the entire workflow, from molecular design to *in vivo* application. The synthesized nanoparticles accumulate in PSMA-positive tumors after systemic administration, as confirmed by thermal imaging. Upon 808 nm laser irradiation, the localized photothermal conversion induces effective tumor heating and ablation at the cellular level. This platform integrates diagnostic and therapeutic functions into a single nanosystem with molecular specificity.

These structural and physicochemical features establish a robust foundation for subsequent photothermal and biological evaluations, enabling the precise delivery of heat to prostate tumors with high selectivity and minimal off-target effects.

### 3.2 *In vitro* photothermal conversion performance of PSMA-OSP_12_ NPs

**Figure 2:**
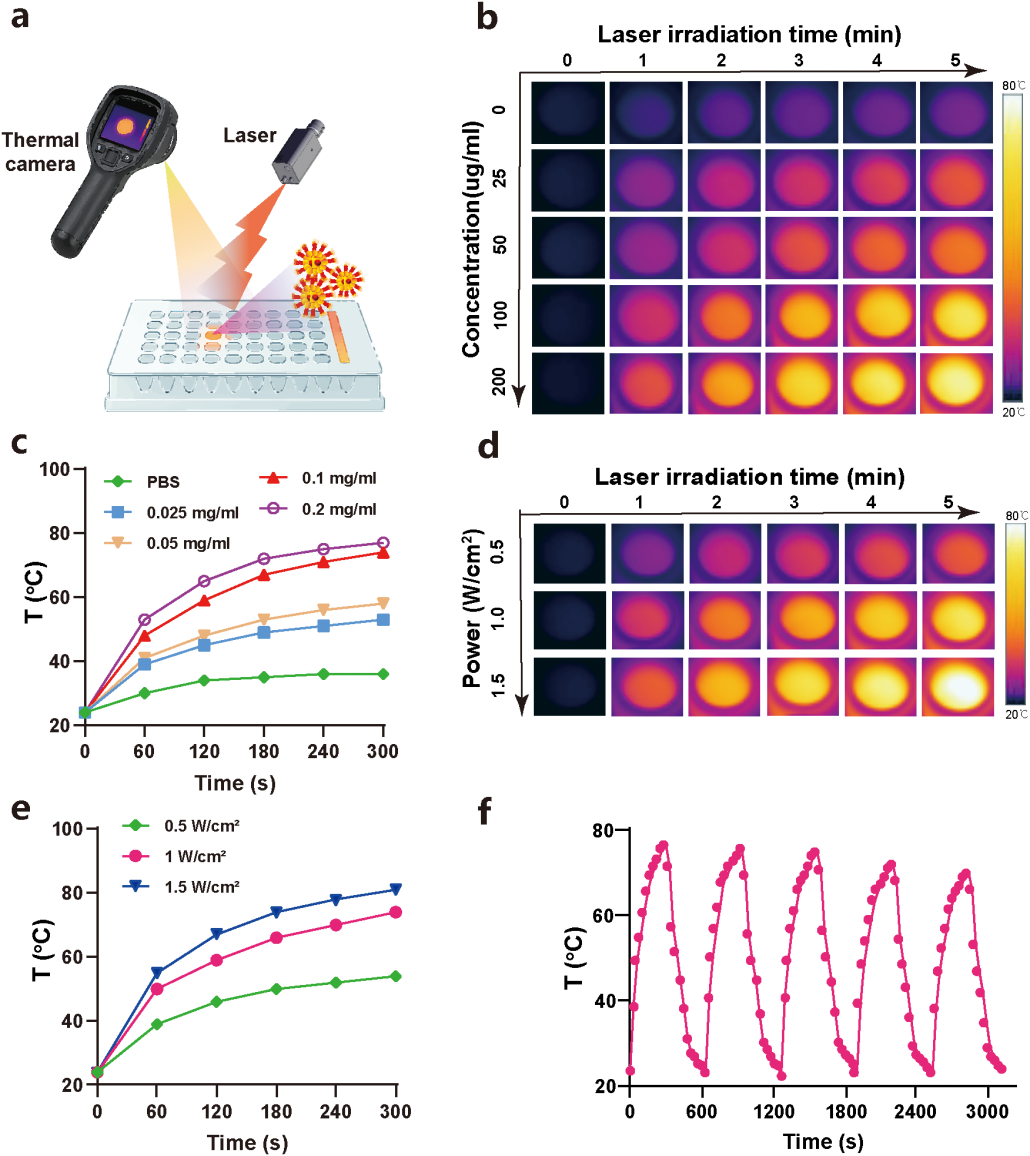
Photothermal conversion performance of PSMA-OSP_12_ NPs *in vitro*. **(a)** Schematic of photothermal performance evaluation. **(b, c)** IR thermal images and temperature rise curves of PSMA-OSP_12_ NPs at various concentrations (0–0.2 mg/mL) under laser irradiation (1.0 W/cm^2^, 5 min). **(d, e)** IR thermal images and corresponding temperature profiles of PSMA-OSP_12_ NPs under different laser power densities (0.5–1.5 W/cm^2^) at a fixed concentration (0.1 mg/mL). **(f)** Photothermal stability of PSMA-OSP_12_ NPs upon five laser on/off cycles.

To evaluate the photothermal responsiveness of PSMA-OSP_12_ NPs under NIR-II excitation, we conducted a series of *in vitro* photothermal tests using an 808 nm laser. As illustrated in the experimental schematic (**Figure 2a**), aqueous dispersions of PSMA-OSP_12_ NPs at varying concentrations were subjected to laser irradiation (1.0 W/cm^2^) while surface temperature changes were monitored using an infrared thermal camera.

The thermal images clearly showed a concentration-dependent temperature elevation (**Figure 2b**). After 5 min of laser exposure, the solution temperature reached 77.3°C at 0.2 mg/mL, compared to 53.4°C at 0.025 mg/mL and 36.2°C change in the PBS control group. The corresponding temperature-time plots (**Figure 2c**) confirmed that the temperature increased rapidly within the first 2 min and plateaued thereafter, demonstrating effective light-to-heat conversion at clinically relevant irradiation parameters.

We further assessed power-dependent performance by exposing PSMA-OSP_12_ NPs dispersions (0.1 mg/mL) to different laser powers (0.5–1.5 W/cm^2^). As shown in **Figures 2d, e**, temperature rose proportionally with increased laser power, reaching up to 80.9°C at 1.5 W/cm^2^. This power- and concentration-responsive heating behavior confirms the tunable thermal output of the system.

Importantly, PSMA-OSP_12_ NPs exhibited excellent photothermal stability, as evidenced by five consecutive on/off laser irradiation cycles (**Figure 2f**). The maximum temperature achieved during each cycle remained consistent (Fluctuation <3°C), with no noticeable photobleaching or degradation observed, supporting long-term utility for repeated therapeutic sessions.

Collectively, these results demonstrate that PSMA-OSP_12_ NPs possess outstanding NIR-II photothermal conversion capabilities, with rapid, controllable, and repeatable heat generation under biologically relevant irradiation conditions—laying the foundation for subsequent *in vitro* and *in vivo* therapeutic evaluations.

### 3.3 PSMA is highly expressed in prostate cancer and serves as an effective targeting site

**Figure 3:**
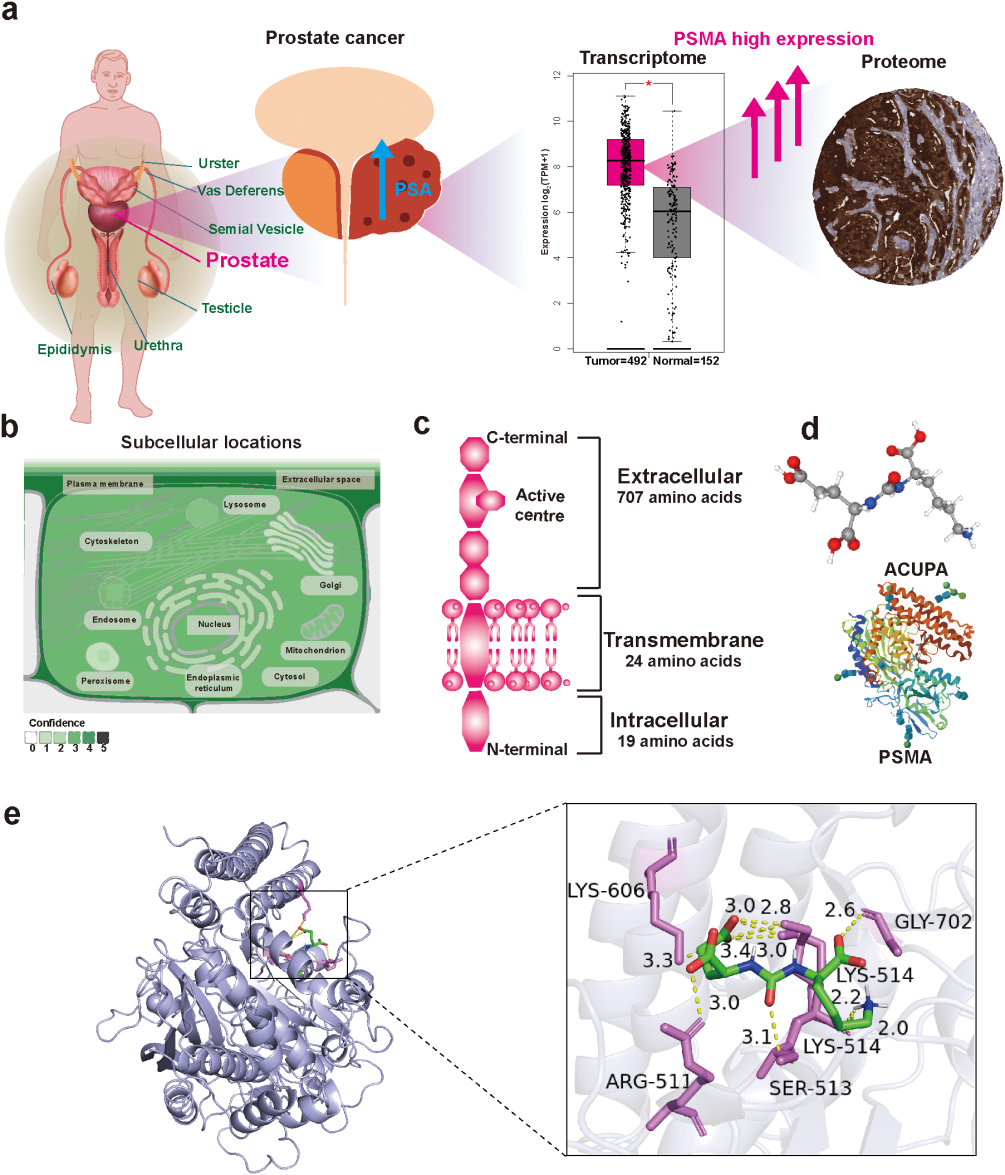
PSMA expression and molecular docking with the targeting ligand. **(a)** Transcriptomic and proteomic analyses reveal high FOLH1 (PSMA) expression in prostate cancer. **(b)** Subcellular localization indicates predominant distribution of PSMA on the plasma membrane and in extracellular regions. **(c)** PSMA protein contains 19 intracellular, 24 transmembrane, and 707 extracellular amino acid residues. **(d)** 3D molecular structures of the PSMA receptor and its ligand ACUPA. **(e)** Molecular docking shows multiple hydrogen bonds between ACUPA and key residues, suggesting strong and stable binding at the active site.

To validate the molecular targeting strategy, we first investigated the expression profile of PSMA (encoded by the FOLH1) in prostate cancer at both transcriptomic and proteomic levels. Data extracted from The Cancer Genome Atlas (TCGA-PRAD) revealed that FOLH1 was significantly upregulated in prostate tumor tissues compared to normal prostate tissues, supporting its role as a tumor-specific surface biomarker (**Figure 3a**). Consistent with mRNA data, immunohistochemical staining results from the Human Protein Atlas confirmed strong PSMA expression in prostate cancer epithelium, while normal prostate tissues showed minimal staining.

Further subcellular localization analysis indicated that PSMA is predominantly expressed on the plasma membrane and extracellular region of prostate cancer cells (**Figure 3b**), making it highly accessible to circulating ligands. Structurally, the full-length PSMA protein consists of three domains: a short 19-amino-acid intracellular segment, a 24-amino-acid transmembrane helix, and a large 707-amino-acid extracellular domain that contains its enzymatic active site and ligand binding pocket (**Figure 3c**). This structural feature allows for efficient recognition by high-affinity ligands under systemic circulation.

To confirm the interaction mechanism, we performed molecular docking simulations between the PSMA extracellular domain and the ACUPA ligand, which was incorporated on the surface of our nanoparticles (**Figure 3d**). The results revealed multiple hydrogen bonds formed between ACUPA and key PSMA residues, including ARG-511, LYS-514, SER-513, and GLY-702 (**Figure 3e**), with bond lengths ranging from 2.6 to 3.2 Å. These residues are spatially clustered around the PSMA catalytic site, suggesting strong electrostatic and hydrogen bonding interactions. The calculated binding energy was −9.2 kcal/mol, indicating a strong and thermodynamically favorable interaction between ACUPA and the PSMA catalytic domain. Importantly, the ACUPA binding site did not interfere with the catalytic pocket, preserving potential enzyme function and enhancing ligand accessibility.

Taken together, these bioinformatic and structural findings establish PSMA as a rational and highly accessible surface marker for targeted prostate cancer therapy. The successful ligand docking further confirms the specificity and stability of the ACUPA-PSMA interaction, supporting its use in engineering targeted nanomedicines with high tumor selectivity.

### 3.4 *In vitro* photothermal cytotoxicity and PSMA-mediated killing specificity

**Figure 4:**
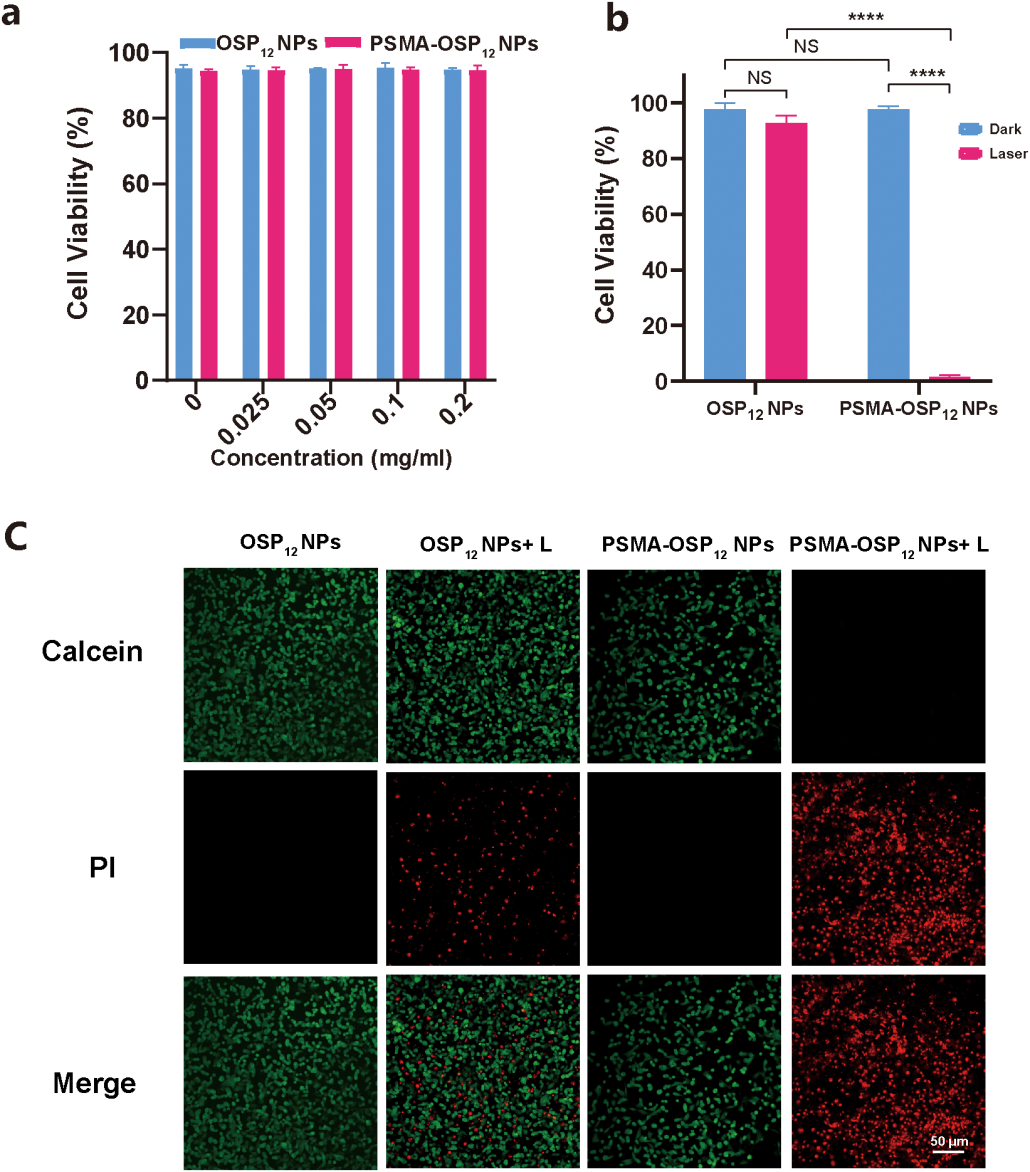
*In vitro* photothermal cytotoxicity of PSMA-OSP_12_ NPs in LNCaP cells. **(a)** Cell viability assays show that both OSP_12_ NPs and PSMA-OSP_12_ NPs are biocompatible without laser irradiation. **(b)** Upon 808 nm laser exposure (1.0 W/cm^2^, 5 min), PSMA-OSP_12_ NPs significantly reduces cell viability compared to non-targeted OSP_12_ NPs. **(c)** Live/dead cell staining using Calcein-AM (green) and PI (red) confirms that only the PSMA-OSP_12_ NPs + laser group shows widespread cell death. Scale bar: 50 μm. “****” indicates p < 0.0001; “NS” indicates no significance.

To assess the biocompatibility and photothermal-induced cytotoxicity of the developed nanoparticles, we conducted comprehensive *in vitro* experiments using PSMA-positive LNCaP prostate cancer cells. As shown in **Figure 4a**, both OSP_12_ NPs and PSMA-OSP_12_ NPs exhibited minimal cytotoxicity across a range of concentrations (0–0.2 mg/mL) in the absence of laser irradiation. Cell viability remained above 95% in all treatment groups, indicating excellent biocompatibility under physiological conditions. These results confirm that the nanoparticles are safe and well-tolerated without external excitation.

We next evaluated the photothermal therapeutic efficacy of each treatment group under 808 nm laser irradiation (1.0 W/cm^2^, 5 min). As shown in **Figure 4b** and the corresponding quantitative data, negligible cytotoxicity was observed in the OSP_12_ NPs, and PSMA-OSP_12_ NPs groups without laser exposure, with cell viabilities consistently above 97%. In contrast, significant cell death was observed only in the PSMA-OSP_12_ NPs + laser group, where viability dropped to as low as 1.49 ± 0.96%, clearly indicating efficient and specific photothermal ablation.

To further confirm the targeting and killing efficiency, we performed Calcein-AM/PI live/dead staining (**Figure 4c**). Consistent with the viability assay, all control groups displayed predominantly green fluorescence, indicative of live cells. Red fluorescence (PI-positive) was minimally detected in the PBS + laser and OSP_12_ NPs + laser groups, suggesting poor photothermal effect in the absence of targeting. However, the PSMA-OSP_12_ NPs + laser group exhibited extensive red staining and nearly complete loss of green fluorescence, highlighting the enhanced cell-killing capacity enabled by PSMA-mediated targeting and NIR-induced thermal response.

Together, these results demonstrate that PSMA-OSP_12_ NPs possess excellent photothermal cytotoxicity with high tumor selectivity. The dramatic difference in therapeutic outcome between targeted and non-targeted formulations underscores the essential role of PSMA-directed delivery in achieving efficient prostate cancer cell eradication under NIR irradiation.

### 3.5 *In vivo* photothermal therapeutic efficacy of PSMA-OSP_12_ NPs in prostate tumor models

**Figure 5:**
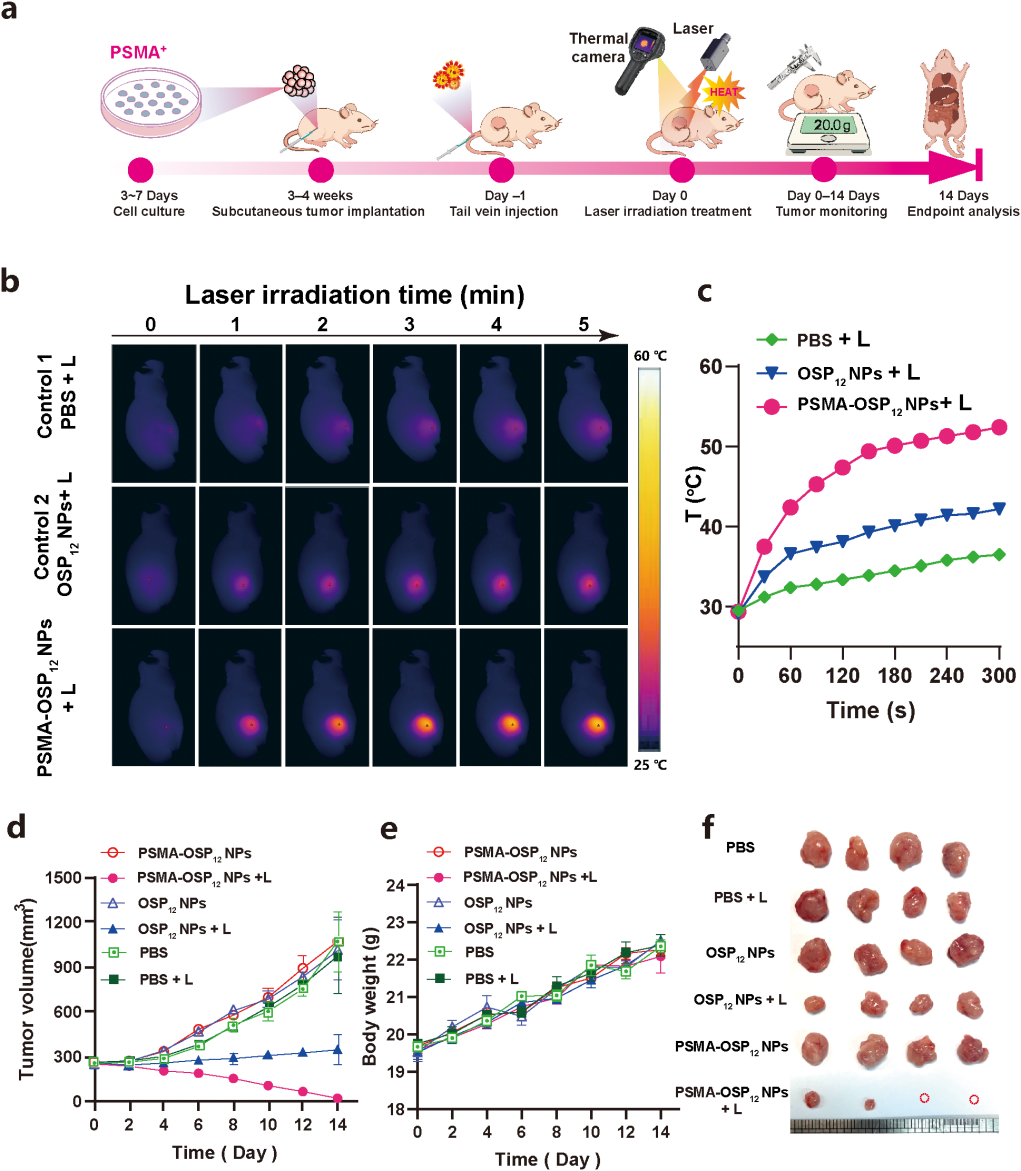
*In vivo* photothermal therapeutic efficacy of PSMA-OSP_12_ NPs in a 22Rv1 prostate tumor model. **(a)** Schematic diagram of the *in vivo* therapeutic protocol. **(b)** IR thermal images show that PSMA-OSP_12_ NPs induces the most pronounced tumor heating under 808 nm laser irradiation (1.0 W/cm^2^, 5 min). **(c)** Tumor temperature curves during laser irradiation. **(d)** Tumor growth profiles over 14 days reveal that the PSMA-OSP_12_ NPs + laser group achieved the greatest tumor inhibition. **(e)** Body weight changes indicate good systemic safety during treatment. **(f)** Photographs of excised tumors on Day 14 confirm the superior antitumor efficacy of PSMA-OSP_12_ NPs -mediated PTT.

To verify the *in vivo* photothermal therapeutic performance of PSMA-OSP_12_ NPs, we established subcutaneous 22Rv1 prostate cancer xenografts in BALB/c nude mice and conducted a multi-stage treatment and evaluation protocol (**Figure 5a**). Once tumors reached ~300 mm³, mice were intravenously injected with PBS, OSP_12_ NPs, or PSMA-OSP_12_ NPs (200 μL of 1 mg/ml). After 24 h post-injection, the tumor regions were irradiated using an 808 nm laser (1.0 W/cm^2^, 5 min), and real-time temperature changes were recorded via infrared thermal imaging.

As shown in **Figures 5b, c**, the PSMA-OSP_12_ NPs + laser group exhibited the most significant temperature elevation, reaching over 52.4°C within 5 minutes of irradiation, while OSP_12_ NPs + laser and PBS + laser groups only achieved 42.2°C and 36.5°C, respectively. This disparity highlights the tumor-specific accumulation enabled by PSMA-mediated targeting, leading to enhanced photothermal conversion *in vivo*.

Tumor volume progression was monitored over the 14-day treatment period as the primary indicator of therapeutic efficacy (**Figure 5d**). The PSMA-OSP_12_ NPs + laser group showed near-complete tumor growth inhibition, with tumor volume decreasing to 18.0 ± 0.96 mm³ by Day 14. In contrast, all other groups displayed continuous tumor growth, with the PBS group reaching1152 ± 87.5 mm³, and the OSP_12_ NPs + laser group only moderately suppressing growth to 726 ± 66.2 mm³. These results underscore the superior therapeutic efficacy of PSMA-OSP_12_ NPs under NIR irradiation, attributable to enhanced intratumoral accumulation and efficient heat generation.

To evaluate systemic safety, we monitored body weight changes throughout the treatment period (**Figure 5e**). All groups maintained stable weight trajectories, with no significant deviation observed, indicating negligible systemic toxicity. On Day 14, all mice were sacrificed, and tumors were excised for direct comparison (**Figure 5f**). Tumors from the PSMA-OSP_12_ NPs + laser group were significantly smaller and less vascularized than those from control groups, visually confirming the effective tumor ablation induced by targeted PTT.

Collectively, these results demonstrate that PSMA-OSP_12_ NPs achieve highly efficient and selective photothermal treatment of prostate cancer *in vivo*, with excellent biosafety and strong tumor suppression in NIR-irradiated settings.

### 3.6 Systemic biosafety evaluation of PSMA-OSP_12_ NPs therapy

**Figure 6:**
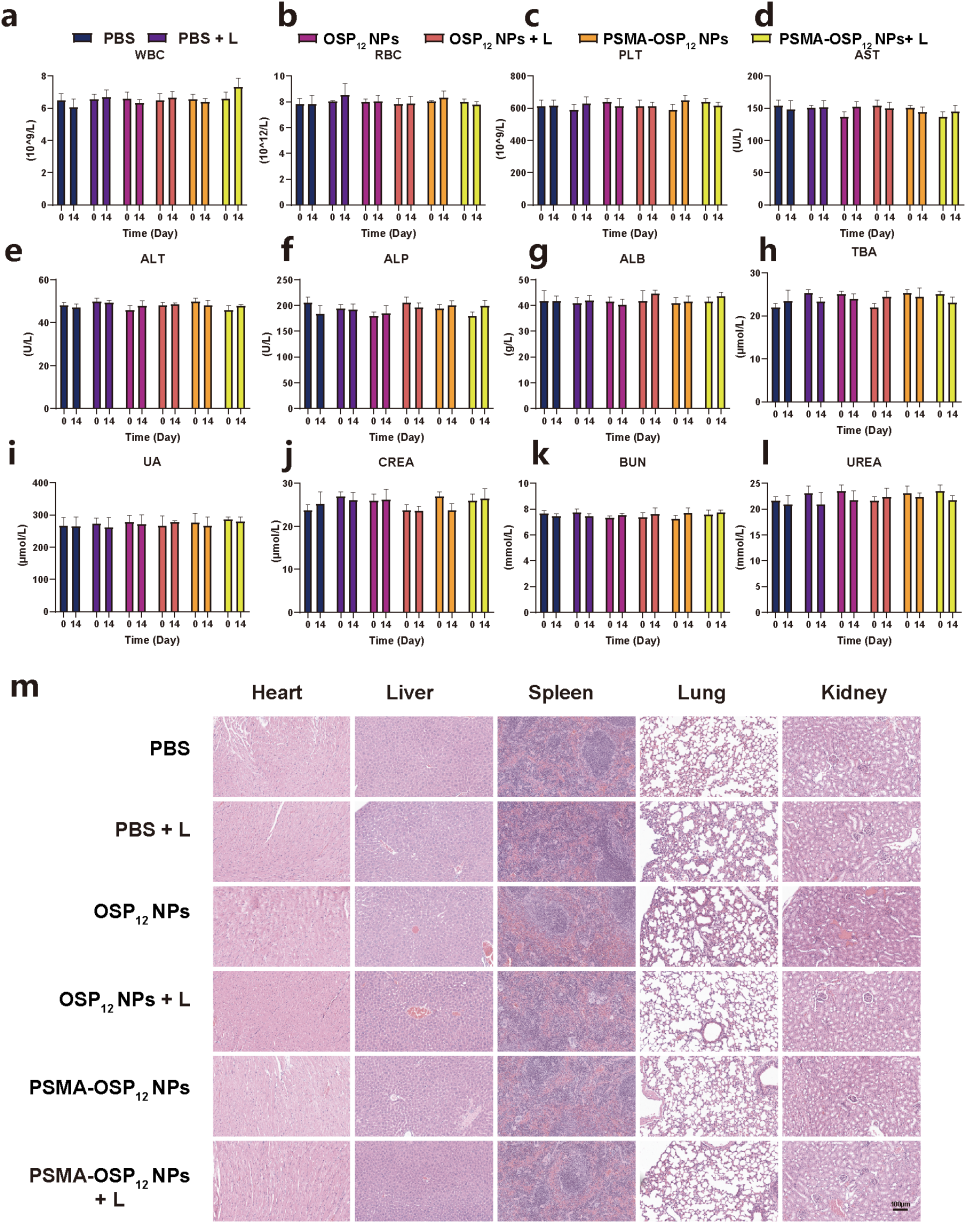
*In vivo* biocompatibility evaluation of nanoplatforms. **(a–c)** Hematological parameters including white blood cell (WBC) counts **(a)**, red blood cell (RBC) counts **(b)**, and platelet (PLT) counts **(c)** at Day 14 post-treatment. **(d–l)** Serum biochemical parameters reflecting liver and kidney function, including uric acid (UA) **(d)**, creatinine (CREA) **(e)**, urea **(f)**, blood urea nitrogen (BUN) **(g)**, aspartate aminotransferase (AST) **(h)**, alkaline phosphatase (ALP) **(i)**, alanine aminotransferase (ALT) **(j)**, albumin (ALB) **(k)**, and total bile acid (TBA) **(l)**. No significant differences were observed among the PBS, PBS+L, OSP_12_ NPs, OSP_12_ NPs +L, PSMA-OSP_12_ NPs, and PSMA-OSP_12_ NPs +L groups (n = 4 per group). **(m)** Representative H&E staining images of major organs (heart, liver, spleen, lung, and kidney) collected at Day 14. Scale bar: 100 μm.

To evaluate the *in vivo* biocompatibility of the nanoplatforms, hematological and biochemical analyses were performed at 14 days post-treatment. As shown in **Figures 6a–c**, key hematological parameters, including white blood cells (WBC), red blood cells (RBC), and platelets (PLT), showed no significant differences among the PBS, PBS+L, OSP_12_ NPs, OSP_12_ NPs +L, PSMA-OSP_12_ NPs, and PSMA-OSP_12_ NPs +L groups. All values remained within normal physiological ranges, indicating that the treatments did not induce hematological toxicity or immune-related abnormalities.

Hepatic function was evaluated by measuring serum levels of alanine aminotransferase (ALT), aspartate aminotransferase (AST), alkaline phosphatase (ALP), albumin (ALB), and total bile acid (TBA) (**Figures 6d–h**). These liver-associated biomarkers showed no significant alterations among the groups, suggesting the absence of hepatocellular injury or hepatic dysfunction induced by the nanoplatforms. Renal function was assessed based on the levels of uric acid (UA), creatinine (CREA), urea (UREA), and blood urea nitrogen (BUN) (**Figures 6i–l**). All values were comparable across groups and fell within the normal range, indicating that the nanoplatforms did not impair glomerular filtration or renal tubular function. To further validate the biosafety, major organs — heart, liver, spleen, lung, and kidney — were subjected to H&E staining (**Figure 6m**). Histological examinations revealed intact tissue architecture without evidence of inflammation, necrosis, hemorrhage, or other pathological changes. The tissue morphology in the treatment groups was indistinguishable from that of the PBS control.

Collectively, these results demonstrate the excellent systemic biosafety and biocompatibility of PSMA-OSP_12_ NPs, indicating their potential for safe clinical translation in targeted photothermal cancer therapy.

## 4 Discussion

PSMA has been widely recognized as a clinically relevant biomarker and therapeutic target in prostate cancer due to its restricted expression in normal tissues and high overexpression in malignant lesions (40, 42, 43). Previous clinical advances in radioligand therapy and PSMA-targeted imaging have validated its translational value; however, conventional probes based on PET or NIR-I fluorophores remain constrained by limited tissue penetration, radiation exposure, and insufficient real-time surgical utility (43–45). In our earlier work, we developed PSMA-OSP_12_ NPs and demonstrated their excellent tumor-targeting specificity and NIR-II fluorescence emission for molecular imaging and intraoperative guidance (25). These findings provided a strong rationale for extending their application beyond diagnosis toward therapeutic intervention.

In the present study, we established PSMA-OSP_12_ NPs as an effective photothermal nanoplatform for prostate cancer ablation. OSP_12_ offers superior photophysical characteristics, including robust NIR-II emission, remarkable photostability, and high photothermal conversion efficiency under 808 nm excitation, while conjugation with ACUPA ensures precise recognition of PSMA-expressing tumor cells. Comprehensive *in vitro* and *in vivo* evaluations confirmed that PSMA-OSP_12_ NPs accumulate selectively in PSMA-positive tumors, achieve rapid and controllable temperature elevation upon laser irradiation, and induce potent photothermal ablation with minimal systemic toxicity. High-temperature PTT ablates but risks collateral injury, while mild-temperature PTT can trigger HSP-driven thermotolerance; ACUPA–PSMA targeting confines heat to tumors, improving the safety–efficacy trade-off. Complementing PSMA, emerging targets—UBE2S, TSPAN18, and PTBP1—offer avenues to combine PSMA-targeted NIR-II PTT with pathway-specific therapeutics to curb metastasis and treatment resistance (46–48).

In addition to achieving direct tumor ablation, PTT provides a controllable thermal modality capable of inducing localized and predictable thermal injury within tumors (49). Compared with non-targeted photothermal agents, PSMA-mediated molecular recognition markedly improves intratumoral accumulation of nanoparticles and restricts heat deposition specifically within PSMA-expressing lesions. This spatial precision reduces off-target thermal damage and enhances overall treatment safety, which is particularly important for prostate cancer given its close anatomical relationship with the urethra, neurovascular bundles, and adjacent organs (50). Furthermore, NIR-II–mediated PTT affords deeper tissue penetration and improved energy delivery compared with traditional NIR-I systems, broadening its applicability for clinically relevant tumor depths (51). The combination of targeted delivery, high photothermal conversion efficiency, and deep-penetrating NIR-II excitation underscores the translational potential of PSMA-OSP_12_ NPs as a precise and minimally invasive therapeutic strategy for prostate cancer. Future studies will focus on validating their performance in orthotopic and metastatic models, as well as evaluating compatibility with clinical NIR-II optical platforms.

Another important consideration for clinical translation lies in biosafety and clearance (14, 52). Our data confirmed that PSMA-OSP_12_ NPs were well tolerated, with no significant hematological or biochemical abnormalities and negligible histopathological changes in major organs. Biodistribution and excretion analyses further indicated hepatobiliary clearance, consistent with previously reported organic semiconducting polymer nanoplatforms (25). These findings mitigate concerns of long-term retention and systemic toxicity, strengthening the translational potential of this material. Nonetheless, further studies are warranted to evaluate long-term immunotoxicity, pharmacokinetics in large-animal models, and compatibility with clinically approved NIR-II imaging systems.

Taken together, this work highlights the continuity and advancement of our research program—from demonstrating the tumor-targeting and imaging performance of PSMA-OSP_12_ NPs in our previous study to establishing their therapeutic efficacy in the present work. By integrating tumor molecular specificity, optical superiority, and potent photothermal activity, PSMA-OSP_12_ NPs represent a versatile nanoplatform that bridges diagnostic and therapeutic applications. Future efforts will focus on elucidating the detailed biological responses induced by PSMA-targeted NIR-II photothermal therapy and exploring rational combinations with systemic therapies to achieve durable tumor control.

Despite these strengths, a key limitation is the sole use of a subcutaneous xenograft model, which cannot fully recapitulate the native prostate tumor microenvironment or metastatic progression. Guided by the 3R principles and our prior confirmation of PSMA-positive versus PSMA-negative differences, we avoided redundant control experiments in this study; nevertheless, future work will incorporate orthotopic and bone metastasis models to strengthen translational relevance.

## 5 Conclusion

In summary, we demonstrated that PSMA-OSP_12_ nanoparticles combine precise tumor targeting, strong NIR-II fluorescence emission, and high photothermal conversion efficiency to achieve effective and safe ablation of PSMA-positive prostate cancer. Building upon our previous imaging-focused work, this study extends the application of PSMA-OSP_12_ NPs to therapeutic intervention and confirms their value as a targeted NIR-II photothermal nanoplatform. Future investigations will further clarify the biological mechanisms associated with PSMA-targeted PTT and evaluate rational combination strategies to enhance long-term tumor control. Collectively, these findings highlight PSMA-OSP_12_ NPs as a promising candidate for advancing precision theranostics in prostate cancer.

## Data Availability

The original contributions presented in the study are included in the article/supplementary material, further inquiries can be directed to the corresponding author/s.
